# Intentional weighting: a basic principle in cognitive control

**DOI:** 10.1007/s00426-012-0435-y

**Published:** 2012-04-12

**Authors:** Jiska Memelink, Bernhard Hommel

**Affiliations:** Institute for Psychological Research and Leiden Institute for Brain and Cognition, Department of Psychology, Cognitive Psychology Unit, Leiden University, Wassenaarseweg 52, 2333 AK Leiden, The Netherlands

## Abstract

Human perception and action are tailored to the situation at hand, and thus reflect the current intentions of the perceiver/actor. We suggest that this is achieved by an “intentional-weighting” mechanism. It operates on the cognitive representations of the features of perceived events and produced event—perceptions and actions that is. Intention- or goal-related feature dimensions are weighted more strongly, so that feature values defined on the respective dimension have a stronger impact on information processing, and stimulus and response selection in particular. This article discusses what intentional weighting is, how such a mechanism may work, and how it relates to available research on attention, action planning, and executive control.

## Cognitive control

In everyday life all kinds of behavior are performed seemingly seamlessly. We are able to plan and execute an enormous variety of actions, such as visiting a friend for having a cup of coffee. This involves arranging transportation to get to your friend’s place, paying attention to traffic on your way over, controlling your motor system to move the cup of coffee to your mouth, and so on. We constantly need to adjust our intended actions to the situation at hand. That is, in our intended action to go over to our friend’s house we need to take into consideration the constantly changing traffic situation in order not to get into an accident. In our intended action to drink coffee we need to adjust our arm and hand position in such a way that enables us to grasp the cup and bring it to our mouth. This flexibility to adjust to the situational context requires some kind of cognitive-control mechanism.

Cognitive control pertains to input as well as to output. That is, we need a control mechanism to select those perceptual events that are important to us and we need a control mechanism to select the actions we want to perform. Since Donders’ (1869) seminal studies, cognitive control is commonly considered to operate online by intervening between stimulus-driven processes and the production of actions (Monsell & Driver, 2000; Norman & Shallice, 1986; Posner & Snyder, 1975; Shiffrin & Schneider, 1977). Accordingly, researchers assume that attentional mechanisms take care of the available stimulus information by prioritizing relevant over irrelevant stimuli were stimulus features, whereas response-selection mechanisms make sure that the most appropriate action is being selected. However, as elaborated elsewhere (Hommel, 2002, 2009), online control mechanisms often operate automatically in the sense that the outcome of their operation can be predicted on the basis of the stimulus being presented in the instructions given to the subject. This means that the true control operations—the processes responsible for cognitive adaptations and flexibility—take place before the first stimulus is being presented and the first response being selected. In other words, the control of input and output control must rely on off-line operations and it is these operations the present article is dealing with. As we will argue, preparing for a task is associated with specific processes that are configuring the cognitive system and the available representations of stimuli and responses in such a way that online control operations can run off more or less automatically. Before we go into the specifics of how this preparation may work, let us consider the cognitive representations the respective processes operate on: the representations of perceptual and action events.

## The Theory of Event Coding

Perceptual and action events are somehow represented in our cognitive system—the sounds we hear, objects we see, and actions we perform. Neuroscientific findings suggest that events are represented in a distributed fashion, as activations in dedicated feature maps spread throughout the cortex (e.g., DeYoe & Van Essen, 1988; Wickens, Hyland, & Anson, 1994). Accordingly, the representation of an event—whether it is perceived or actively produced—can be considered a network of distributed codes that represent the features of the event. How these distributed codes might be operated on to generate perception and action is being discussed by the Theory of Event Coding (TEC) suggested by Hommel, Müsseler, Aschersleben, and Prinz (2001a, 2001b).

TEC is a general framework explaining how perceived and produced events (i.e., stimuli and actions) are cognitively represented and how their representations interact to generate perception and action. TEC claims that perception and action features are coded in a common format and assumes that perception, attention, intention, and action share and operate on a common representational domain (Prinz, 1990). This notion implies that perceiving an object and acting upon that object is essentially the same process and involves the same network of represented features. Accordingly, perception may influence action, and vice versa—as indeed demonstrated in numerous studies (e.g., Craighero, Fadiga, Rizzolatti, & Umiltà, 1999; Hamilton, Wolpert, & Frith, 2004; Knoblich & Flach, 2001; Proffitt, 2006; Schubö, Aschersleben, & Prinz, 2001).

The theoretical move that allows TEC to relate perception to action, and vice versa, is grounded in the ideomotor assumption that actions are cognitively represented in terms of codes of their perceptual effects (for recent reviews, see Hommel, 2009; Shin, Proctor, & Capaldi, 2010). In a nutshell, the idea is that agents are continuously registering the perceptual consequences of their movements and integrating the representations of these consequences with the motor patterns that brought them about (Lotze, 1852; James, 1890). If so, action-effect representations become effective retrieval cues of the actions that are likely to produce the represented effects, which provides the knowledge base necessary to anticipate and actively produce action effects—that is, voluntary action control.

According to TEC, voluntary action is thus more than producing motor outflow, as it entails the active anticipation of a wanted perceptual outcome (the action goal), the actual production of that outcome, and the repeated integration of that outcome with the action producing it. By the same token, TEC has a very active concept of perception, as it considers perceptual input as a consequence of actively seeking and producing this input. Indeed, we are unable to perceive a visual object without having oriented our body and moved our eyes toward this object, unable to sense its surface without systematically touching it, and so forth. Hence, both perception and action are sensorimotor processes that need to integrate sensory information with the movements responsible for bringing them about. The only difference between what researchers call perception and what they call action control is that analyzing the former focuses on the processing of the actually produced sensory information, while analyzing the latter focuses on the selection of motor output based on the anticipation of this sensory consequences.

## The intentional-weighting mechanism

Considering that both perceived events and actively produced events are cognitively represented in terms of their sensory features raises the possibility that the processing of these events is controlled in similar ways and perhaps even by the same mechanisms. Indeed, Hommel et al. (2001a) have speculated that the representations of both types of events may be mediated and contextualized through an “intentional-weighting” mechanism. Before we present a generalized version of this weighting mechanism, let us consider two concrete examples to explain the basic logic underlying it.

First, consider a simple action, such as grasping a bottle of water. The successful planning of such an action requires quite a bit of knowledge about grasping in general and grasping water or other bottles in particular. According to the ideomotor principle, the agent selects the grasping action by representing the intended action effect, for instance by imagining holding the bottle in his or her dominant hand. However, in an experienced agent, the motor pattern producing a grasp is likely to be associated not only with representations of grasped water bottles but also with representations of all sorts of other objects that have been and can be grasped: toys, fruits, tools, and so on. At the same token, the representation of the grasped water bottle is likely to be associated not only with grasping actions but also with other action that could result in holding a water bottle, such as catching. Hence, having acquired and being able to recall and reactivate particular action-effect associations is insufficient to tailor the cognitive representations to the task at hand. What is needed is a mechanism that restricts the possible action opportunities to grasping and the possible action outcomes to water bottles, thereby selecting the task-relevant action and action-effect representation. How can that be achieved?

The central claim that we want to defend in the following is that such selections are the consequence of changing the weights of features that are coded on task-relevant dimensions: intentional weighting. When preparing for a task, so we suggest, retrieving or forming an intention automatically increases the weight of features coded on those dimensions that have been experienced, or are assumed to be, and/or actually are necessary for coding task-relevant stimuli and responses. This assumption is common to models of visual search, where preparing for a task is suspected to involve the priming of task-relevant feature dimensions, such as color or shape or any other target-defining feature (e.g., Wolfe, 1994), or even higher-order perceptual or semantic features (e.g., Barsalou, 1999; Pecher, Zeelenberg, & Raaijmakers, 1998). Priming a feature dimension is assumed to increase the impact of features being coded on it on object selection and performance. As the entire dimension receives more weight, all features defined on it will be more salient. Note that the same logic can be applied to action selection. If participants are asked to discriminate between left and right keypresses or between approach and avoidance movements of a joystick, say, this necessarily renders the perceptual dimensions that are coding for the discriminative action feature relevant: horizontal location and the forward–backward (i.e., depth) dimension, respectively. Indeed, according to the ideomotor principle, action selection considers the actions’ perceptual consequences, which suggests that it relies on the perceptual dimensions on which the task-relevant consequences are defined. This suggests that preparing for executing the same motor pattern can imply the intentional weighting of very different perceptual dimensions, depending on which of the perceptual consequences of the pattern are actually intended.

To summarize, we claim that preparing for the perception and the production of an event includes the automatic priming of task-relevant feature dimensions (and/or increasing the output gain of feature values coded on these dimensions), which increases the impact of codes represented on this dimension on information processing. Features that are task-relevant thus receive more weight than irrelevant features, which increases the probability that those features dominate the cognitive representation of the whole event. The very same perceptual event and motor pattern may thus be cognitively coded in very different ways. Intentional weighting is assumed to occur in, and affect perception as well as action. The weighting of features in perception may be called “attentional” weighting since it affects the way attentional processes operate. Nevertheless, we claim that the weighting processes are not any different from the weighting processes that are affecting action selection, which is why we summarize and relate both types of weighting by referring to “intentional weighting”—so to indicate that the weighting processes are a direct consequence of the current intention to perceive and to act. We assume that the off-line, preparatory intentional weighting serves to control and enable online control processes, such as attentional selection or action selection, which then can be carried out more or less automatically. In other words, we assume that off-line control can automatize online control (Hommel, 2002, 2007, 2009). As we will emphasize below, the assumed off-line nature of intentional weighting implies that it is a slow and time-demanding process that creates control configurations that are inert and thus take time to modify or deactivate. In the following, we will review various studies from different fields of research that shed more light on the intentional-weighting mechanism and that provide evidence for the claim that intentional weighting is a general principle underlying cognitive control or, perhaps better, cognitive meta-control.

## Intentional weighting in perceptual search

Strong evidence for the operation of a weighting mechanism is provided by observations in visual search tasks. In visual search tasks participants are to detect a target item among non-target items (the distractors) that do or do not share features with the target. We can distinguish between feature search tasks and conjunction search tasks. In feature search tasks, the target that needs to be detected has a unique feature that none of the distractors have, such as a different color or shape than the other items. This unique feature results in the target to pop out. In conjunction search, the target is not defined by a single unique visual feature but by a combination of two or more features, therefore the information of several features must be integrated to locate the target.

According to the feature integration theory by Treisman (1999) and Treisman and Gelade (1980), basic features are represented in feature maps. Treisman distinguishes two kinds of visual search tasks, feature search and conjunction search. Feature search can be performed fast and pre-attentively for targets defined by primitive features. Conjunction search is the serial search for targets defined by a conjunction of basic features. It is much slower and requires attention. According to Treisman visual attention is needed to bind basic features together so that the conjunction of features (a target that has a unique combination of features which by themselves are not unique) can be computed, or objects can be recognized. As this process is assumed to be serial it is therefore a much slower process than feature search. Wolfe (1994) and Wolfe, Cave, and Franzel (1989) extended the feature integration model of Treisman into what is known as the Guided Search model. In this model basic features are processed in parallel. The initial processing of features produces output maps that integrate bottom-up saliency (local differences between feature values) and top-down expectations, with attention being drawn to locations with particularly high levels of activation.

Targets that are not sharing any features with the non-targets are called singletons, which are known to ‘pop out’ and, thus, easy to detect. In contrast to claims that singleton pop out is entirely stimulus driven (Theeuwes, Reimann, & Mortier, 2006), a number of findings suggest contributions from a top-down mechanism that operates by weighting task-related feature dimensions more strongly (Found & Müller, 1996; Müller, Heller, & Ziegler, 1995). In their 1996 studies Found and Müller (1996) observed that repeating the target-defining dimension in a pop-out search task produces a repetition benefit. Interestingly, there was little or no benefit when repeating the target-defining features compared to changing the features within a dimension. Thus, the benefit was primarily due to dimension-specific repetition, indicating a passive stimulus-driven dimension weighting system that merely tracks the defining dimension and assigns weight to the target-defining dimension (Found & Müller, 1996).

Müller, Reimann, and Krummenacher (2003) investigated whether weightings in visual search can be modified intentionally. They had participants search for pop-out targets. Prior to the pop-out detection search task, the subjects were cued the likely dimension of the upcoming target, being either color or orientation. This dimensional cuing facilitated their response. In an additional experiment the likelihood of the upcoming feature was cued, such as a specific color. In the latter experiment no facilitating effect was found. However when a particular feature was cued, there was a dimension-specific cuing effect. When no cue was given or neutral cues were presented, then the system tuned into the dimension defining the target on that trial. On subsequent trials, there was a slight benefit for the dimension the system was tuned into on trial N-1, showing that dimensional cueing spills over to the next trial. These experiments show that cuing a whole dimension (such as color) modulates visual search whereas cuing specific features (such as the color red) does not have that effect, again showing the dimension-specific nature of the weighting mechanism. Their findings indicate that even early visual processes can be modulated in a top-down fashion, by intentional knowledge-based processes that facilitate target detection. The cueing process assigns more weight to the cued dimension, therefore facilitating detection of features defined on that dimension. In this dimension-weighting account, it is argued that weight is allocated to the various dimensions and that an attended dimension (e.g. the dimension color or the dimension shape) temporarily receives more weight and hence facilitates the pop out of a singleton defined on the attended dimension. Furthermore, the spillover effects indicate that dimensional weighting is a slow or sticky process, or needs to be overruled by a new cue, hence more weight being put on another dimension.

**Fig. 1:**
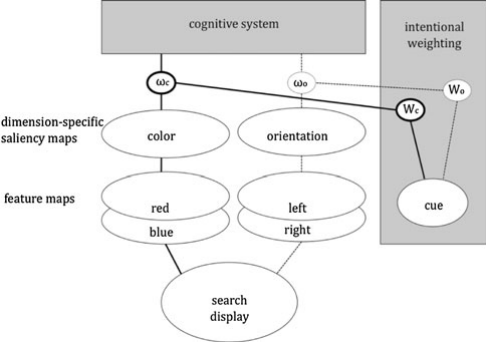
Intentional weighting in visual processing. Selection of a visual event is a function of two factors: the bottom-up saliency of particular features of the event (coded on dimension-specific saliency maps) and the top-down weighting of feature dimensions (operationalized here by determining the output gain of information coded on saliency maps; see Wykowska et al., 2009). Bottom-up saliency and top-down weighting interact to determine the impact of a feature value coded on a given dimension on further processing

The observations of Müller et al. (2003) show that dimension weighting plays a role in visual attention: Cuing a feature dimension apparently increases the saliency of stimuli coded on this dimension, and this improves performance even if this dimension does not need to be reported. This fits with our assumption that intentional weighting operates on all feature dimensions that are involved in discriminating between task-relevant and task-irrelevant stimuli and/or responses. The intentional-weighting mechanism for visual search is sketched in Fig. 1, where the weighting of different dimensions is influenced by bottom-up as well as top-down factors. Although this example scenario refers to the detection of simple features, Weidner and Müller (2009) have recently shown that and how dimensional-weighting logic can be extended to conjunction search.

Visual search tasks are commonly assumed to tap into stimulus-selection processes only. However, more recent observations suggest that stimulus- and response-selection processes interact—just as the TEC would suggest. For instance, Bekkering and Neggers (2002) investigated how action intentions influence visual selection processes in a visual search task, in which subjects either grasped or pointed at a target. Targets varied in color and orientation. The authors measured saccadic eye movements, since saccadic eye movements precede goal-directed aiming movements, whether one grasps or points to a target, which allows measuring effects on action planning but not action execution. Saccades toward orientation-defined targets were more accurate in the grasping than in the pointing condition, while no effect of manual action was found for color-defined target objects. Since object orientation is relevant for grasping while color is not, the authors suggest that planning a particular motor action modulates the visual processing of that stimulus. It thus seems that action intentions can modify the tuning of neural channels representing specific stimulus features. This fits with the idea that intentions can assign more weight to a dimension that is relevant for performing that action—an issue we will elaborate in the following section.

## Intentional weighting in action planning

Since action events and perceptual events rely on a common coding system, it follows that dimensional weighting should not only apply to perceived events but also to action events as well (Hommel, 2004; Hommel et al., 2001a). Furthermore, since TEC does not distinguish between feature codes for perception and feature codes for action, making a particular dimension relevant for perceptual discriminations should automatically induce task relevance of the same dimension in action discriminations (cf. Hommel, 2005). Indeed, converging evidence suggests that planning an action primes perceptual dimensions related to that action, that is, dimensions that provide information for discriminating between action alternatives and/or for specifying parameters of the action.

Traditionally, action planning has been investigated separately from perceptual processes, as it was assumed that perception and action planning are modular processes that are separated in time, function, and mechanisms. However, increasing evidence suggests that action planning is affected by perceptual processes and, more importantly for the present discussion, perceptual processes are systematically affected by action planning (e.g., Craighero et al., 1999; Hamilton, Joyce, Flanagan, Frith, & Wolpert, 2007; Müsseler & Hommel, 1997a; Wohlschläger, 2000; for an overview, see Schütz-Bosbach & Prinz, 2007).

Research that has looked into the question of how action planning affects perception has initially focused on the feature level, that is, on whether particular actions facilitate or impair the perception of particular action-related features. For instance, it has been shown that an object-grasping action can be initiated more quickly if it is signaled by a go signal that has the same shape as the object to be grasped (Craighero et al., 1999). More direct evidence for the sharing of feature codes between perception and action was provided by Müsseler and Hommel (1997a) and Hommel and Müsseler (2006) (see also Milliken & Lupiáñez, 2007; Oriet, Stevanovski, & Jolicoeur, 2007), who observed that planning an action with particular spatial features tends to prevent the planning individual from perceiving the same feature in other, action-independent visual events (for an overview, see Hommel, 2004). This suggests that planning an action involves the *activation* of codes referring to the features of the action, which facilitates the detection of action-related information (as in Craighero et al., 1999), as well as the *binding* of these codes into a coherent action plan, which impairs the creation of other bindings in perception (Müsseler & Hommel, 1997b) or action planning (Stoet & Hommel, 1999) to the degree that they feature-overlap.

More recent research has provided evidence that perception and action interact not only at the feature level but also at the level of feature dimensions, which speaks more directly to our present discussion. For instance, in the study of Fagioli, Hommel, and Schubotz (2007), participants were preparing a grasping or pointing action before searching for a shape- or location-defined visual target. It turned out that preparing for a grasping action facilitated the search for shape targets, whereas preparing for a pointing action facilitated the search for location targets. This suggests that preparing for an action induces a stronger weighting of feature dimensions that provide action-relevant information, such as shape features for grasping and location features for pointing action (Hommel, 2010). A follow-up study of Fagioli, Ferlazzo, and Hommel (2007) showed that this effect does not require active planning but can also be produced by priming the action representation through videos showing the action. This suggests that action representations contain information about the feature dimensions that provide action-relevant information and that this information automatically primes action-related perceptual dimensions whenever the action representation is activated. Interestingly, people seem to make use of this association between action representations and related feature codes even in what seems like purely perceptual tasks. For instance, Schubotz and von Cramon (2001, 2002, 2003) reported that monitoring a series of visual events for a feature-defined oddball induces activation in action-specific area of the premotor cortex. In particular, attending to shape targets active areas involved in grasping actions, while attending to location targets activates areas involved in pointing.

**Fig. 2:**
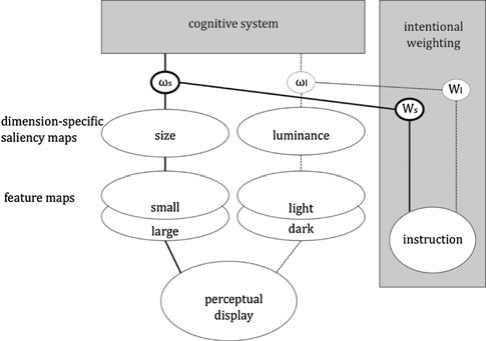
Intentional weighting in action planning. Preparing for an action increases the weight of feature dimensions (i.e., increases the output gain of feature values coded on that dimension) that provide information that is suited to specify open parameters of the action, such as size in the case of a grasping action or location or luminance in the case of a pointing action (see Hommel, 2010). As in the case of visual attention, top-down weighting interacts with bottom-up saliency to determine the impact of a feature value from a particular dimension on further processing

Further converging evidence for the impact of action planning on target detection comes from Wykowska and colleagues. In the study of Wykowska, Schubö, and Hommel (2009), subjects prepared for either a grasping movement or a pointing movement but were not to execute the movement until a visual search detection task had been performed. While being prepared for the grasping or pointing movement subjects performed a visual search detection task in which a target circle had to be detected by its luminance or by its size. After completing the detection task, subjects were to execute the prepared movement. It turned out that preparing for a grasping action facilitated the search for shape targets, whereas preparing for a pointing action facilitated the search for luminance targets. These findings suggest a more general effect of action planning on selection processes, showing that even early perceptual processes in visual search can be influenced by an action accompanying it. Moreover, the findings reinforce the idea that preparing for an action primes an entire dimension, thereby enhancing all features defined on that dimension. To summarize, increasing evidence suggests that preparing for a particular type of action involves a readjustment of weights in such a way that action-related perceptual dimensions will receive more weight—as indicated in the scenario shown in Fig. 2.

## Intentional weighting and task switching

We have now seen that the intentional-weighting mechanism may help us understand the way perception and action planning interact. In particular, considering the logic and operation characteristics of weighting mechanisms it is easy to see that and why action intentions and action planning affect visual search and bias the processing of feature-overlapping stimuli. However, as we will show in the following section, intentional weighting also affects other layers of behavioral control. As we have pointed out in the introduction, intentional-weighting mechanisms are assumed to operate off-line and they are likely to be slow and to produce relatively “sticky” control configurations. How sticky these configurations can be is obvious from studies on task switching.

Task-switching paradigms are investigating the limits of behavioral flexibility. They commonly require participants to switch between two or more tasks with the main interest being whether performance on switch trials is worse than on trials in which the task is repeated. Numerous studies provided strong evidence that switching between tasks indeed produces performance costs, that is, performance is worse on switch trials than on repeat trials (for an overview, see Monsell, 2003; Kiesel et al., 2010). Most interestingly for our purposes, switching costs are obtained even if participants have full pre-knowledge about which task to perform and ample time to prepare—the so-called residual switching costs (e.g., Meiran, 1996). Numerous factors have been suggested to contribute to residual switching costs (see Kiesel et al., 2010) and one of them is presumably related to intentional weighting.

As Allport, Styles, and Hsieh (1994) have considered, at least part of the performance deficit after a task switch may be due to proactive interference, that is, to the fact that the previous task representation is still sufficiently activated to compete with the current task representation. To investigate the impact of proactive interference, Meiran, Chorev, and Sapir (2000) have manipulated the interval between the previous response and the point in time the cue is informing the participant about the upcoming task. The longer this interval was the smaller were the residual task-switching costs (note, however, that this observation does not seem to generalize across all display formats: Proctor, Koch, Vu, & Yamaguchi, 2008). Meiran et al. (2000) considered the possibility that such a decrease reflects the gradual deactivation of the previous task representation, and they suggested that switching between tasks might require the adjustment of weights assigned to particular stimulus and response features. In their experimental task, participants were to press one of two diagonally arranged buttons (say, a top-left and a bottom-right button) in response to either the horizontal or vertical location of visual stimuli that randomly appeared in one corner of a 2 × 2 grid. As suggested by Meiran et al. (2000), preparing for the horizontal task might require a stronger weighting of stimulus and response features related to the horizontal location dimension, whereas the vertical task requires a readjustment of these weightings so to increase the weight of the vertical features. This possibility would fit with the observation that residual switching costs are strongly reduced, if not eliminated, if the response sets of the two tasks do not overlap (Kieffaber, Kruschke, Walker, & Hetrick, 2012; Yeung & Monsell, 2003). Hence, there are reasons to assume that intentional weighting, and the after-effects of previous weightings, contribute to performance costs under task-switch conditions.

More direct evidence for a contribution of weighting mechanisms would be provided by demonstrating not only non-specific performance decrements, when weightings have to be modified, but also specific biases of control processes induced by previous weightings. We sought for this kind of evidence by employing a two-dimensional Simon task (Simon & Rudell, 1967). The Simon task is a choice reaction time task in which the location of the stimulus is irrelevant while the location of the response is not, like when participants press a left versus right key in response to the color of stimuli that randomly appear on the left or right of a display. The common finding is that responses are performed faster and more accurately when response location and stimulus location correspond, despite the fact that no relationship between the two exists—the Simon effect (Simon & Rudell, 1967; see reviews by Lu & Proctor, 1995; Hommel, 2011).

Memelink and Hommel (2005, 2006) turned the basic setup into a two-dimensional Simon task. Like in the task used by Meiran (1996), the response buttons were diagonally arranged (e.g., top-left and bottom-right) and the stimuli appeared at the corners of an imaginary 2 × 2 grid. Importantly, this setup allows for the separate computation of two types of Simon effects: a horizontal effect (comparing performance when the horizontal location of stimulus and response did or did not match) and a vertical effect (comparing performance when the vertical location of stimulus and response did or did not match). Participants were to switch between this two-dimensional Simon task and another, logically unrelated task that required paying attention to either the horizontal or the vertical location of visual stimuli. As expected, switching from the horizontal version of the attention task increased the size of the horizontal Simon effect, whereas switching from the vertical version of the attention task increased the size of the vertical Simon effect. This provides direct evidence that preparing for the attention task involved the adjustment of weighting for horizontal and vertical stimulus features and that these weightings carried over to the Simon task, where they apparently affected both stimulus and response coding. Hence, attending to the horizontal stimulus dimension in the attention task seems to have turned the top-left key press in the Simon task into a “left” rather than a “top” event, while attending to the vertical stimulus dimension in the attention task apparently did the opposite.

**Fig. 3:**
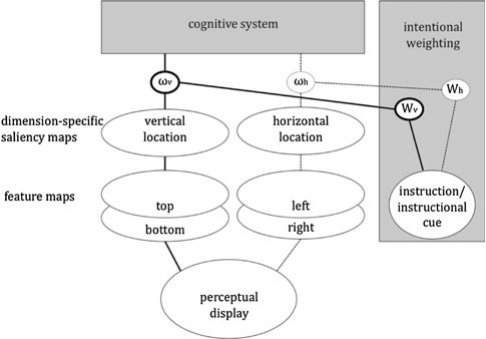
Intentional weighting in task switching. The task set defines task-relevant feature dimensions for perceptual and action-related decision-making. Depending on the task, this may affect feature weighting in a top-down fashion, very much like an action control (see Fig. 2), or in a bottom-up fashion, as with task cues that are correlated with the task relevance of a particular feature dimensions. The weightings interact with bottom-up saliency to determine the impact of feature values coded on a particular feature dimension to determine their impact on further processing

Another study that suggests that feature weightings can carry over from one task to another was reported by Meiran, Hommel, Bibi, and Lev (2002). They investigated task-switching performance in a task version that measured whether participants felt sufficiently prepared to carry out a new task (Dixon, 1981; Dixon & Just 1986). Participants were presented with cues that identified the upcoming task and could take as much time as they wanted to prepare. Whenever they felt ready, they indicated this by pressing a “ready” button. It was expected that longer preparation time would indicate better preparation, which should result in a negative correlation between preparation time and the reaction time in the upcoming task. In contrast, however, this correlation was positive, suggesting that longer preparation was associated with worse performance. The authors considered that participants might not have been able to consciously access the degree of their preparation, which rendered the readiness response and the subsequent task-specific response more or less unrelated. However, people are likely to vary in concentration over time, which might have induced the positive correlation: if a participant would happen to be highly focused on the task, this would be likely to speed up both the readiness response and the task-specific response a few seconds later, but if a participant would happen to be distracted during the readiness interval, he or she would also be likely to be distracted during the actual task trial. In other words, the values of global cognitive-control parameters might vary spontaneously over time, so that temporarily close responses would be equally affected. This interpretation was supported by a second experiment, in which task readiness was directly manipulated. Two preparedness conditions were introduced: a high-readiness condition, in which the instruction was to indicate readiness when completely ready, and a low-readiness condition, in which participants were to indicate readiness as quickly as possible. Results were as in the first experiment, that is, long reaction times in the readiness task yielded long reaction times in the task-switching task. This provides strong support for an off-line operating intentional-weighting mechanism: if response speed receives more weight in one task, this setting is likely to spill over to other, temporarily close tasks and responses. Figure 3 provides an illustration of this scenario.

## Intentional weighting of action effects

Up to now we have looked into the impact of intentional weighting on perceptual features and on action features, as well as interactions between them. According to TEC, the cognitive representations of action features derive from the perceptual effects the respective actions are known to produce. Pressing a left or right key creates perceptual events that, among other things, have the feature of occurring on the left or right side of some reference point, or from each other. Accordingly, the cognitive representations of left and right keypresses should include codes referring to the leftness and rightness of these events, which renders the actions “left” and “right” in cognitive terms. As we have seen, emphasizing particular spatial dimensions in one task can spill over to other tasks and modulate the spatial characteristics of the cognitive representation of actions (e.g., by emphasizing or de-emphasizing their leftness and rightness). An interesting, counter-intuitive feature of TEC is that it does not distinguish between action features that seem to be more intrinsic, such as the leftness or rightness of a particular finger movement, and more extrinsic, overlearned consequences of an action, such as the tone that is produced by pressing a particular piano key. Accordingly, it should be possible to affect and bias the cognitive representation of actions not only through the intentional weighting of intrinsic action effects, such as the spatial location or endpoint of a movement, but also through the weighting of extrinsic, acquired action effects.

A study that speaks of the possibility to modify the cognitive representation of action by drawing attention to particular extrinsic action effects is that of Hommel (1993). He had participants carry out an auditory Simon task, in which they pressed left and right keys in response of the high or low pitch of a tone that was randomly presented through a left or right loudspeaker. Pressing a key produced a light flash on the opposite side, so that pressing the left key had a visual effect on the right side and pressing the right key had an effect on the left side. When participants were instructed to “press the left/right key” in response to the tone, a standard Simon effect was obtained: performance was better if the tone appeared on the same side where the correct response key was located. This suggests that the actions were spatially coded with respect to the location of the key or the finger operating it. However, when participants were instructed to “flash the right/left light” in response to the tone, the Simon effect was reversed: now performance was better if the tone appeared on the side where the visual action effect was expected. This suggests that the actions were spatially coded with respect to the visual action effect, whose cognitive code was in this case apparently weighted more strongly than the cognitive code of the location of the response key or the finger.

**Fig. 4:**
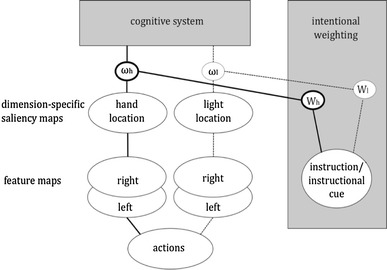
Intentional weighting in action-effect coding. Effectors, and action effects are spatially coded and the relative weights these codes receive depend on the instruction in the task representation. In the example, the location of the hand is presented to matter more than the location of some experimental light effect of the action (see Hommel, 1993). If hand location and light locations differ, instruction determines the stimulus–response compatibility

The resulting scenario is shown in Fig. 4, where the weighting of different response dimensions is influenced by intention. That is, focus on the response key gives the spatial features of the keypress response dimension more weight while focus on the visual effect gives more weight to the more remote action effect.

## Conclusion

We have seen that several cognitive phenomena can be explained in terms of an intentional-weighting mechanism. As the available research suggests, the intentional-weighting principle is applicable to, and provides a better understanding of selection processes in (visual) perception, action, executive control, and the dynamic interplay between perception and action. As such, the principle seems to grasp the very essence of the contextual flexibility and adaptivity of human behavior.

The intentional-weighting mechanism seems to work in such a way that weights are assigned to whole dimensions/domains such as color or location, rather than to specific feature values such as ‘red’ or ‘up’. Activation (or putting more weight on a domain) results in a greater impact of feature values coded on this domain or dimension in subsequent cognitive operations. Changing these weightings and transferring them from one domain to another seems to be a rather slow process, which invites cross-talk effects and task-switching costs.

Given the broad generality of the intentional-weighting mechanism and its applicability to the various cognitive phenomena, it is interesting to ask whether and to which degree these phenomena are actually different. From the perspective of the Theory of Event Coding (Hommel et al., 2001a), selecting a stimulus is not any different from selecting a response: both are considered events of the same sort that are cognitively represented alike, so that selecting a stimulus in selecting a response differs with respect to the consequences of the selection but not regarding the selection operation. Accordingly, it may very well be that the top-down contributions to such selection operations are of the same kind as well. Recent work of Rangelov, Müller, and Zehetleitner (2012) indicates that the logic underlying the proposed weighting mechanism can also be extended to task-set-dependent processes that intervene between stimulus and response. Although Rangelov et al. (2012) argue for multiple weighting systems, the operational characteristics of their weighting mechanism is very similar to the one described here. A similar recent development that is, however, more devoted to the interaction between perception and action is the Multidimensional Vector Model of Stimulus–Response Compatibility suggested by Yamaguchi and Proctor (2012). The model allows for representing differential weightings of stimulus dimensions as a function of response properties—which the authors demonstrate to account for a number of stimulus–response compatibility phenomena and which perfectly fits the theoretical approach we suggest. In any case, the suggested principle of intentional weighting is not only rather general but also sufficiently simple to further reduce the loans of intelligence taken when explaining intentional human behavior.
